# Sinapic Acid Ameliorates Oxidative Stress, Inflammation, and Apoptosis in Acute Doxorubicin-Induced Cardiotoxicity via the NF-*κ*B-Mediated Pathway

**DOI:** 10.1155/2020/3921796

**Published:** 2020-03-10

**Authors:** Yousef A. Bin Jardan, Mushtaq Ahmad Ansari, Mohammad Raish, Khalid M. Alkharfy, Abdul Ahad, Fahad I. Al-Jenoobi, Nazrul Haq, Mohd Rashid Khan, Ajaz Ahmad

**Affiliations:** ^1^Department of Pharmaceutics, College of Pharmacy, King Saud University, Riyadh 11451, Saudi Arabia; ^2^Department Pharmacology and Toxicology, College of Pharmacy, King Saud University, Riyadh 11451, Saudi Arabia; ^3^Department Clinical Pharmacy, College of Pharmacy, King Saud University, Riyadh 11451, Saudi Arabia

## Abstract

In the present study, we explored SA's activity against DOX-induced cardiotoxicity and revealed its underlying mechanisms. Male Wistar rats (weight, 190-210g; *n* = 6) were randomly divided into four groups: group I, normal control; group II, DOX 15 mg/kg via intraperitoneal (ip) route; group III, administered DOX+SA 20 mg/kg; and group IV, administered DOX+captopril (CAP 30 mg/kg). SA and CAP were administered orally for seven days, and DOX (15 mg/kg) was injected intraperitoneally an hour before SA treatment on the fifth day. Forty-eight hours after DOX administration, animals were anesthetized and sacrificed for molecular and histology experiments. SA significantly mitigated the myocardial effects of DOX, and following daily administration, it reduced serum levels of lactate dehydrogenase (LDH) and creatine kinase isoenzyme-MB to near normal values. Levels of oxidative stress markers, glutathione-peroxidase, superoxide dismutase, and catalase, in the cardiac tissue were significantly increased, whereas malondialdehyde levels decreased after SA treatment in DOX-administered rats. Furthermore, DOX caused an inflammatory reaction by elevating the levels of proinflammatory cytokines, tumor necrosis factor-*α* (TNF-*α*), interleukin-1*β* (IL-1*β*), and endothelin- (ET-) 1, as well as nuclear factor kappa-B (NF-*κ*B) expression. Daily administration of SA significantly repressed TNF-*α*, IL-1*β*, ET-1, and NF-*κ*B levels. caspase-3 and Bax expression, bcl-2-like protein and caspase-3 activities and levels. Overall, we found that SA could inhibit DOX-induced cardiotoxicity by inhibiting oxidative stress, inflammation, and apoptotic damage.

## 1. Introduction

Since 1960, doxorubicin (DOX, Adriamycin) has been a potent chemotherapeutic agent. DOX belongs to the class of anthracyclines and is derived from Streptomyces peucetius [1, 2]. This potent anticancer agent is used as a chemotherapy for various malignancies such as lymphomas; leukemia; Kaposi's sarcoma; and breast, gastric, and esophageal carcinomas. However, owing to the lethal cardiotoxicity of DOX, its use is restricted [3]. DOX-induced cardiotoxicity is associated with oxidative stress, apoptosis, and inflammation [4, 5].

Several reports have indicated that DOX induces inflammation in cardiac muscles and vasculature through nuclear factor kappa-B (NF-*κ*B), which is a critical regulator of inflammatory and immunological reactions. DOX is reduced to semiquinone which generates reactive oxygen species (ROS) such as superoxide and hydrogen peroxide [6] and depletes glutathione peroxidase (GPx) and catalase (CAT), thereby reducing the myocardium's ability to eliminate ROS. Furthermore, DOX chelates iron, and the subsequent complex catalyzes the transformation of peroxide radicals to reactive hydroxyl radicals, resulting in oxidative and mitochondrial damages to the myocardium [6, 7]. The potential implication of apoptosis in DOX-induced cardiotoxicity is via the intrinsic apoptotic pathway [4, 8]. In addition, this agent plays a crucial role in chemotherapy but as previously mentioned, cardiotoxicity limits its use. Natural antioxidants have been adopted as a chemoprotective approach to reverse cardiotoxicity [9]. Sinapic acid (SA) is a potent free radical scavenging agent that can inhibit lipid peroxidation and restore endogenous antioxidants [10–12]. Sinapic acid and its cardioprotective role against ischemia/reperfusion, isoprotenol, and arsenic have also been previously reported [13, 14]. Hence, we postulated that SA can efficiently repress DOX-induced cardiotoxicity and sought to explore SA's activity against DOX-induced cardiotoxicity and reveal its underlying mechanisms in the present investigation.

## 2. Materials and Methods

### 2.1. Drugs and Chemicals

SA, DOX, and CAP were procured from Sigma-Aldrich (USA). Antibodies against NF-*κ*B (p65), caspase-3, Bax, Bcl-2, *β*-actin, and HRP secondary antibodies were obtained from Santa Cruz (USA). The NE-PER Extraction Kit was purchased from Pierce Biotechnology (USA). Rat TNF-*α*, IL-1*β*, and myeloperoxidase (MPO) ELISA kits were bought from R&D Systems (USA).

### 2.2. Experimental Design

Adult male Wistar rats (weight, 190-210g) were obtained from the animal facility of King Saud University, Riyadh, Saudi Arabia. The experiment proposal was authorized by the Ethics Committee of the Experimental Animal Care Society, King Saud University, Saudi Arabia (KSU-SE-19-40). Animals were arbitrarily and equally divided into four categories (*n* = 6 animals/group): group I, normal control provided with normal saline; group II, administered DOX 15 mg/kg via intraperitoneal (ip) route; group III, administered DOX+SA 20 mg/kg; and group IV, administered DOX+captopril (CAP; 30 mg/kg). CAP was used as the standard drug for cardiac protection as per earlier reports [15, 16]. SA and CAP were administered orally for seven days, and DOX (15 mg/kg) was injected intraperitoneally an hour before SA treatment on the fifth day. Forty-eight hours after DOX administration, animals were anesthetized with ketamine 100 mg/kg and 10 mg/kg, ip [17]. Blood samples were collected from all groups, and serum separation was carried out at 5000 rpm for 15 min. All rats were sacrificed, and their hearts were harvested for molecular and histopathological examinations.

### 2.3. Serum Biochemical Indices

Lactate dehydrogenase (LDH) and creatinine kinase MB fraction (CK-MB) levels were estimated in the rat serum with commercial calorimetric kits (Merck). Nitric oxide (NO) and endothelin-1 (ET-1) levels were assessed.

### 2.4. Oxidative and Antioxidant Indices

Cardiac tissues were balanced to make a 10% (*w*/*v*) RIPA-buffered homogenate using T 25 Digital ULTRA-TURRAX®. After centrifugation of the homogenate, the upper layer was collected for biochemical analysis. Protein level was quantified by the Lowry method [18]. The level of MDA, in the cardiac tissue, was examined by using a lipid peroxidation (malondialdehyde (MDA)) assay kit (Sigma-Aldrich, St. Louis, MO, USA). Lipid peroxidation was determined by the reaction of MDA with thiobarbituric acid (TBA) to form a colorimetric product, proportional to the MDA present. The intensity of the color was measured spectrophotometrically at 532 nm. Glutathione peroxidase (GPx) activity was estimated by a glutathione peroxidase kit specific for rats, according to the manufacturer's protocol (ZellBio GmbH, Germany). The activity of glutathione peroxidase (GPx) was determined by NADPH-coupled assay according to the method of Lawrence and Burk [19], and this enzyme catalyzes the oxidation of glutathione by hydrogen peroxide. The oxidation of NADPH was followed spectrophotometrically at 340 nm. SOD activity was estimated by Beauchamp and Fridovich [20]. The reaction mixture consisted of 0.5 ml of cardiac PMS, 1 ml of 50 mM sodium carbonate, 0.4 ml of 25 *μ*M NBT, and 0.2 ml of 0.1 mM EDTA. The reaction was initiated by an addition of 0.4 ml of 1 mM hydroxylamine-hydrochloride. The change in absorbance was recorded at 560 nm. Determination of catalase (CAT) activity was examined by a Catalase (CAT) assay kit specific for rats, according to the manufacturer's protocol (abcam. ab83464). Catalase (CAT) level was assessed using H_2_O_2_ as the substrate by the methods of Beers and Sizer [21]. The disappearance of H_2_O_2_ was followed spectrophotometrically at 240 nm.

### 2.5. Cytokine and Inflammatory Marker

TNF-*α*, IL-1*β*, and MPO contents were examined in the cardiac tissue using ELISA kits (R&D Systems). Absorbance was read at 450 nm.

### 2.6. Preparation of Nuclear and Total Protein Extracts

Nuclear and cytosol proteins were isolated with the NE-PER Kit (Pierce Biotechnology). Western blot was carried out as per protocols of Towbin et al. [22]. In brief, 25 *μ*g of protein was transferred to PVDF membranes, blocked in 4% skimmed milk in TBS (1% Tween 20), and incubated at 4°C overnight with the antibodies, cleaved caspase-3, Bax, Bcl-2, NF-*κ*B (p65), I*κ*B*α*, and *β*-actin. This was followed by several washings with 1% Tween and TBS. Incubation was then performed with secondary antibodies for 2 h at room temperature. Bands were observed using Luminata™ Western Chemiluminescent HRP Substrates (Millipore, Billerica, MA, USA). A densitometric analysis of the immunoblots (LI-COR C-Di-Git Blot Scanners (Lincoln, NE, USA)) was also performed.

### 2.7. Histological Analysis

Cardiac tissues were fixed with 10% formalin, dehydrated in a gradient series of alcohol, and embedded in paraffin blocks. Tissues were then sliced into 4 *μ*m sections, stained with hematoxylin and eosin (H&E), and examined under a light microscope.

### 2.8. Statistical Analysis

All data are expressed as mean ± SEM. Statistical analysis was performed using the one-way analysis of variance (ANOVA).

## 3. Results

This section may be divided by subheadings. It should provide a concise and precise description of the experimental results, their interpretation, and the experimental conclusions that can be drawn.

### 3.1. Cardiotoxicity Indices

Although the intake of DOX resulted in a reduction in body weight of treated rats, this alteration was not statistically different from that of normal control rats. Absolute body weight and heart index, however, were significantly reduced by 16.78% and 9.40%, respectively, in the DOX-administered group compared to the corresponding values in the control group. When DOX-administered animals were pretreated with SA and CAP, absolute weight was, respectively, enhanced by 5.23% and 6.83%, whereas heart index was increased by 10.37% and 9.14% compared to those in the control group (Table 1).

### 3.2. Cardiotoxicity Biochemical Indices

DOX-administered rats displayed a significant increase in the levels of LDH (214.13%) and CK-MB (102.14%) compared to those in normal rats. In contrast, animals treated with SA and CAP displayed a significant reduction in these parameters (LDH, 65.33% and 66.25%; and CK-MB, 38.07% and 39.90%) unlike animals in the DOX group (*p* < 0.05; Table 2).

### 3.3. Effects of SA and CAP on NO and ET-1 Levels

ET-1 levels were significantly increased (59.51%; *p* < 0.001), whereas those of NO were significantly reduced (66.76%) in DOX-administered rats compared to those in normal rats. Treating DOX-induced rats with SA and CAP resulted in a significant reduction in ET-1 levels (29.13% and 26.81%, respectively) and a significant increase in NO levels (100.07% and 119.075%, respectively) compared to those in rats treated with DOX alone (Table 3).

### 3.4. Effects of SA on the Levels of Lipid Peroxidation and Antioxidant Enzymes

Lipid peroxidation and antioxidant enzyme activities were measured in all groups (Table 4). DOX was found to induce lipid peroxidation as demonstrated by the increase in MDA level (69.57%) compared to that observed in normal group rats. The SA- and CAP-administered rats demonstrated a significant reduction in MDA levels (63.20% and 65.32% nmol/mg, respectively) compared to those in DOX-induced rats (*p* < 0.01; Table 4). Similarly, the DOX-administered group exhibited a significant decrease in SOD (58.28%), GSH (60.66%), and CAT (66.69%) levels compared to those in the normal group (*p* < 0.01). Treatment with SA and CAP significantly restored the depletion of antioxidant enzymes such as SOD (44.35% and 27.56%; *p* < 0.01, *p* < 0.01), GSH (43.16% and 59.64%; *p* < 0.001, *p* < 0.001), and CAT (112.81% and 112.59%; *p* < 0.001) compared to the levels found in DOX-administered rats (Table 4).

### 3.5. Effects of SA and CAP on the Levels of TNF-*α*, IL-1*β*, and MPO

**Figure 1:**
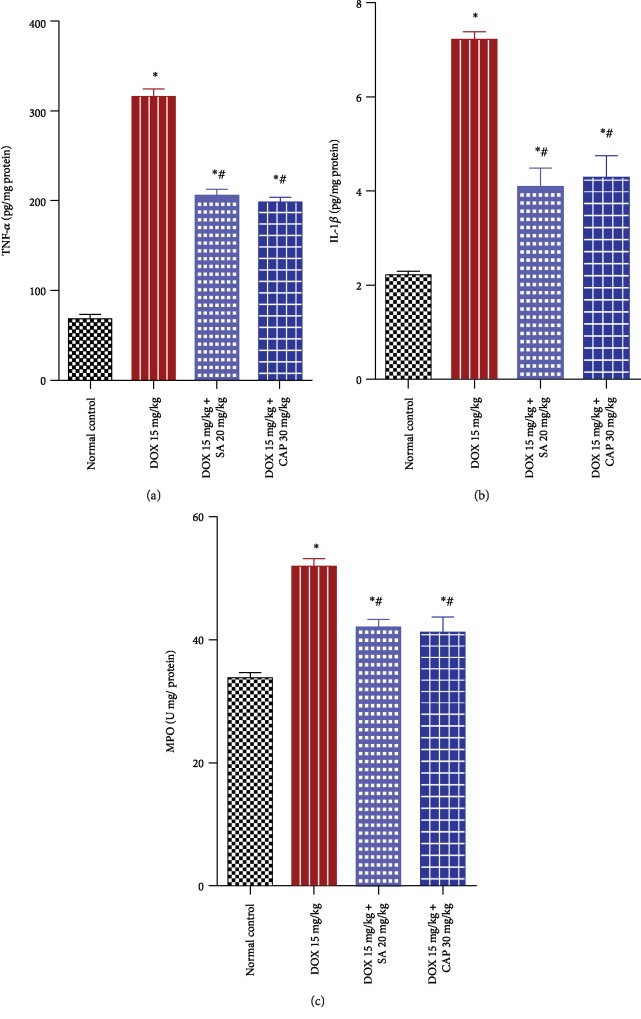
Effect of sinapic acid (SA) on the levels of proinflammatory cytokines (TNF-*α*, IL-1*β*, and MPO) in control and experimental rats. The results are presented as mean ± SEM with six animals per group. ^∗^ denotes significant differences compared to the control group (*p* < 0.05); ^#^ denotes significant differences compared to the DOX group (*p* < 0.05).

The levels of the inflammatory cytokines, TNF-*α* and IL-1*β*, and inflammatory marker, MPO, were determined in the cardiac tissue of all groups as illustrated in Figure 1. DOX-treated rats showed significant increases in TNF-*α* (349.61%), IL-1*β* (228.007%), and MPO (53.98%) levels in this tissue compared to those in normal rats (*p* < 0.01). However, treatment with SA and CAP significantly downregulated the inflammatory responses of TNF-*α* (36.36% and 38.41%), IL-1*β* (43.25% and 40.55%), and MPO (19.08% and 20.62%) compared to the levels observed in DOX-administered rats.

### 3.6. Effects of SA and CAP on the Expression of Bax, Caspase-3, Bcl-2, and NF-*κ*B

**Figure 2:**
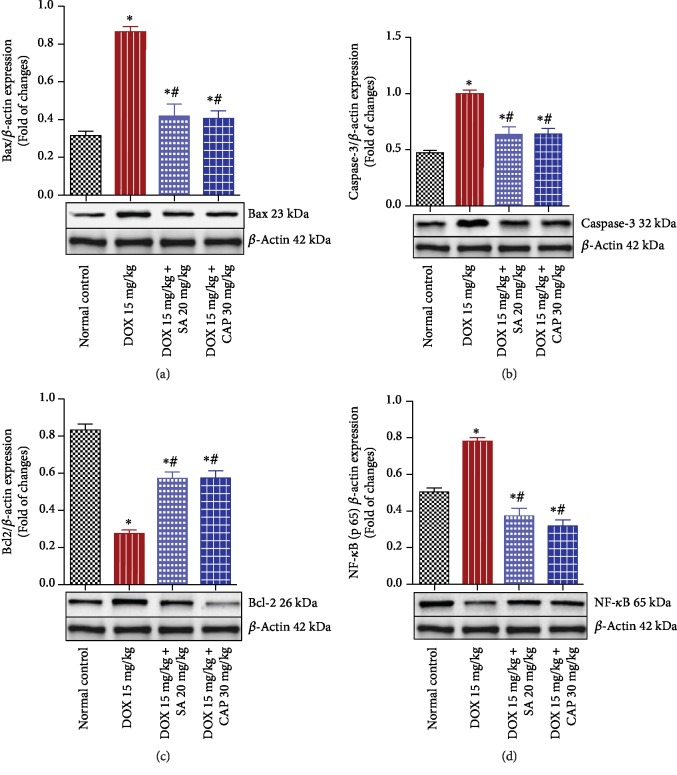
Effect of sinapic acid (SA) on (a) Bax, (b) caspase-3, (c) Bcl-2, and (d) NF-*κ*B protein expression in control and experimental rats. The results are presented as mean ± SEM with six animals per group. ^∗^ denotes significant differences compared to the control group (*p* < 0.05); ^#^ denotes significant differences compared to the DOX group (*p* < 0.05).

Expression of the apoptotic proteins Bax and caspase-3 significantly increased, whereas that of the antiapoptotic protein Bcl-2 significantly reduced in the DOX group compared to that found in the normal group. Pretreatment with SA and CAP significantly enhanced the expression of Bcl2 and reduced the expression of Bax and caspase-3 compared to those observed in the DOX group, thereby reducing the apoptosis-induced cardiac injuries (Figures 2(a)–2(c)). In the DOX group, a relatively significant activation of NF-*κ*B occurred in the cardiac tissue compared to that observed in rats in the normal group. In contrast, pretreating DOX-administered animals with SA and CAP significantly attenuated NF-*κ*B expression (Figure 2(d)).

### 3.7. Histopathological Evaluation

**Figure 3:**
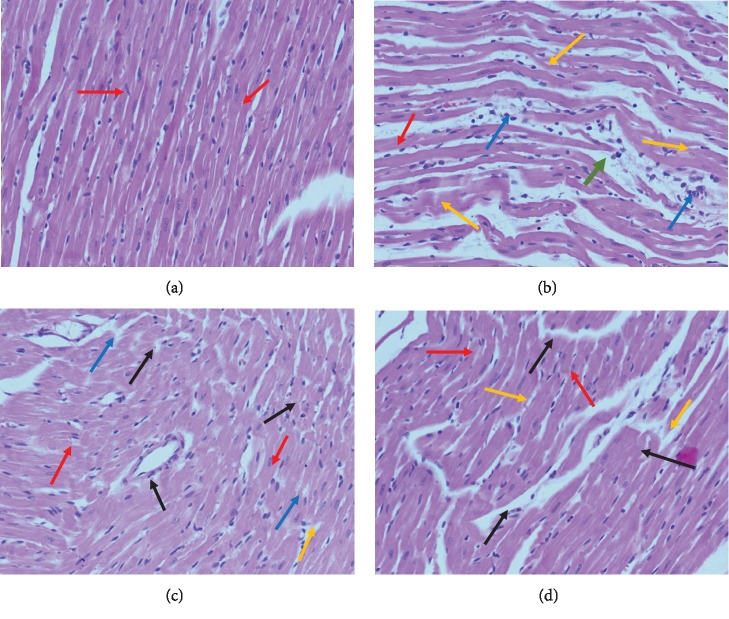
Effect of sinapic acid (SA) pretreatment on the histopathological changes in the cardiac tissue of DOX-treated rats. Photomicrographs of the myocardium tissue in (a) normal rats (group I) exhibiting normal cardio myofibril architecture (red arrow); (b) DOX-treated rats (group II) exhibiting perivascular cuffing (green arrow) of the vasa vasorum with intimal fibrosis, disrupted medial elastic fibers with diffuse interstitial fibrosis, myocytolysis (orange arrow), and myonecrosis (blue arrow); (c) DOX (15 mg/kg)+SA (20 mg/kg)-treated rats (group III) showing decreased degree of myonecrosis (black arrow) and less infiltration of inflammatory cells; and (d) captopril (CAP; 30 mg/kg)+SA (20 mg/kg)-treated rats displaying reversal of myocardial damage based on the reduction in the degree of necrosis and minor infiltration of inflammatory cells. Heart tissues were stained with hematoxylin and eosin and visualized under a light microscope at 100x magnification.

Myocardium tissues from normal rats had regular cell distribution and myocardium architecture (Figure 3(a)). Histological examination of the hearts from DOX-administered animals revealed a significant loss of myofibrils and wavy fibers (Figure 3(b)). Pretreatment with 20 mg/kg of SA and CAP, however, nearly preserved the normal architecture of the myocardium (Figures 3(c) and 3(d)).

## 4. Discussion

DOX is a life-threatening chemotherapeutic agent used for cancer treatment. Because it results in severe cardiotoxicity, its clinical utility is thus limited [1, 23]. DOX induces cardiomyopathy, and if this advances, a poor prognosis occurs, often leading to fatality [24]. Oxidative stress, inflammation, and apoptosis play a significant role in DOX-induced cardiotoxicity [4, 23, 24]. In addition, DOX-induced cardiomyopathy is the primary cause of heart failure. SA possesses potent antioxidant and anti-inflammatory activities; therefore, it is postulated that SA could also have defensive effects against doxorubicin-induced cardiotoxicity. In the present study, we employed a DOX-induced cardiotoxicity rodent model to evaluate the cardioprotective effect of SA in DOX-induced cardiotoxicity using biochemical, morphometric, and histological parameters. No mortality was found in the groups included in the study; however, a reduction in body weight, heart weight, and heart index occurred in the DOX-induced groups. We, then, examined the probable molecular mechanisms underlying the cardioprotective effects of SA. Free radical-induced oxidative stress has been reported as main contributing factors to the DOX-induced deformation of heart tissues [25, 26]. DOX anthracycline structure has been indicated to release enzymatic and nonenzymatic redox cycle liberation of ROS from molecular oxygen [27]. Free radical scavenging, therefore, delivers important ways to protect against DOX-induced oxidative injury. Free radical scavenging, therefore, delivers important ways to protect against DOX-induced oxidative injury. Sinapic acid (SA, 3,5-dimethoxy-4-hydroxycinnamic acid) is a phytoconstituent extensively present in spices, berry fruits citrus, vegetables, oilseed crops, and cereals [28] and is known to possess various pharmacological activities, such as antioxidant, antihypertensive, antimicrobial, anti-inflammatory, antianxiety, and anticancer activities. SA is richly present in plants of the Brassicaceae family [29]. It has potent ROS/RNS- and free radical-scavenging activity to protect against tissue damage [30, 31]. Being its potent antioxidant and anti-inflammatory activities, we hypothesized that SA could also have defensive effects against doxorubicin-induced cardiotoxicity.

Pretreatment with SA and CAP significantly ameliorated the loss in these parameters, reflecting the cardioprotection that they can elicit and align with the results of previous studies [32–34]. CAP is used as a standard cardioprotective agent against DOX-induced cardiotoxicity [15]. The prevailing experimental indication suggests that DOX influences the accumulation of free radicals in cardiac tissue, causing injuries to intracellular components as well as the myocardium and its membranes [2]. Injuries to the myocardial membranes result in the release of LDH and CK-MB in serum or plasma. LDH and CK-MB remain the standard biomarkers for cardiac injuries. Increases in LDH and serum CK-MB levels suggest that DOX causes cardiac injuries in cellular membranes, which result from pretreatment with SA and CAP. This elevation in LDH and CK-MB aligns with that found in previous investigations [5, 23]. The anti-ischemic effect of SA and CAP on cardiac tissues was determined by TTC staining [35]. The DOX-administered groups exhibited a significant increase in % area of necrosis, and this was significant in animals pretreated with SA and CAP.

Present literature suggests that DOX-induced oxidative stress is due to accumulation of free radicals in cardiac tissues. Cardiac tissues are particularly vulnerable to free radicle injuries as they contain low levels of antioxidant enzymes such as SOD, GSH, and CAT. Further, DOX displays high affinity toward phospholipid components of mitochondrial membranes in cardiac cells, leading to its accumulation in heart tissues [1, 6, 36]. DOX-induced mitochondrial injuries are critical as they presumably exhibit life-threatening adverse effects on the heart's contractile function. Therefore, we employed a protocol that would initiate DOX-induced oxidative stress prior to SA and CAP intervention to explore the extent of progressive cardiac damage. CAP has been used as reference standard. Captopril is a potent, competitive inhibitor of angiotensin-converting enzyme (ACE), the enzyme responsible for the conversion of angiotensin I (ATI) to angiotensin II (ATII). ATII regulates blood pressure and is a key component of the renin-angiotensin-aldosterone system (RAAS). Captopril is used to treat high blood pressure (hypertension), congestive heart failure, kidney problems caused by diabetes, and to improve survival after a heart attack [16, 37].

Pretreatment with SA and CAP could minimize DOX-induced cardiotoxicity indices via several routes. Lipid peroxidation (MDA) increased in the cardiac membranes of DOX-administered animals. MDAs are the end products of lipid peroxidation; thiobarbituric acid (TBA) is reacted with MDA, which is resulting in a color compound, which can be determined spectrophotometrically. An elevation in MDA level suggests an enhancement in oxidative stress and a reduction in antioxidant enzymes [6, 38]. In the current investigation, there was a marked elevation in lipid peroxidation and MDA levels and depletion in the antioxidant defenses (SOD, GSH, and CAT) following DOX-induced cardiotoxicity. SA has been reported to exert detrimental effects on lipid peroxidation and oxidative stress, but it is also capable of controlling the elevation in MDA and depletion of antioxidant defenses to moderate extents [12, 39]. SA and CAP pretreatment significantly reduced lipid peroxidation (MDA) and restored the depleted antioxidant enzymes (SOD, GSH, and CAT) following DOX-induced cardiac damage, aligning with the reported literature [5, 23, 34, 40].

DOX induces an inflammatory response in rat myocardium [41] and is believed to induce a series of inflammatory reactions within the myocardium by upregulating NF-*κ*B, inflammatory cytokines (TNF-*α* and IL-*β*), and MPO [42, 43]. Consistent with previous reports, the findings of the current investigation support the primary role of inflammation in the pathogenesis of DOX-induced cardiotoxicity, validating the substantial increase in TNF-*α*, IL-1*β*, and MPO levels in the DOX group compared to those in the normal control group. Although the primary pathway promoting the increases in inflammatory markers is yet to be fully elucidated, this might be caused by impaired antioxidant capacity, increased levels of free radicals, and the existence of lipid peroxidation, which together are initiating factors for the alterations. Certainly, it has been established that elevated levels of inflammatory markers are associated with enhanced oxidative stress, which is known to initiate inflammatory reactions by activating NF-*κ*B, which regulates the release of cytokines [44].

DOX treatment generates ROS, disrupts endothelium-based cardiac myocyte functions, and encourages the release of endothelial cell-derived ET-1, PGI2, NO, and NRG-1, causing cardiac toxicity [45]. Generation of NO leads to peroxynitrite ions that induce nitrostative stress in the myocardium, suppress myocardial contractility, and induce apoptosis [46, 47]. DOX promotes the increase in NF-*κ*B p65, TNF-*α*, and IL-1*β* levels in addition to increasing the ET-1 and NO levels. This reflects oxidative stress induced inflammatory responses. On the contrary, SA and CAP treatment decreased NF-*κ*B expression and inhibited the downstream inflammatory cascade. Therefore, SA and CAP appear to possess potent anti-inflammatory effects. Our data corroborates with our previous studies, where SA was found to be an inhibitor of oxidative stress and inflammatory cytokines [12, 48]. Moreover, NF-*κ*B p65 activation may lead to DOX-induced apoptosis in the myocardium [49]. In the current study, data from western blot analysis indicated that DOX may induce apoptotic and necrotic cellular injuries in the myocardium as indicated by the increase in Bax and caspase-3 protein expression and decrease in the expression of the antiapoptotic protein, Bcl-2. SA and CAP administration significantly downregulated the expression of the proapoptotic proteins, Bax and caspase-3, and upregulated that of the antiapoptotic, Bcl-2. These findings reveal that the antiapoptotic activity, at least in part, is responsible for the cardioprotective effect of SA in DOX-induced cardiotoxicity in rats. These results corroborate with those of previous reports [13, 50, 51].

## 5. Conclusions

The present investigation demonstrates that SA administration can ameliorate DOX-induced cardiotoxicity by suppressing oxidative stress, inflammation, and apoptosis. The potential mechanism of its protective role is mediated by inhibition of inflammation and apoptosis via downregulation of NF-*κ*B. Our findings above suggested that the use of SA could be expected to have synergistic efficacy and significant potential against cardiotoxicity induced by DOX. Thus, the approach of SA could be applied to treat or prevent DOX-induced cardiotoxicity in the future. SA may act as a cardioprotective agent that promotes the safe use of DOX in patients exposed to chemotherapy; further clinical studies were required to prove its clinical efficacy.

## Figures and Tables

**Table 1:** Effects of SA on body weight and heart body weight ratio.

Groups	Initial body weight	Final body weight	Gain in body weight	Heart body weight ratio
Normal control	213.00 ± 1.71	225.20 ± 1.71	12.20 ± 2.19	4.26 ± 0.05
DOX 15 mg/kg	221.80 ± 1.65^∗^	187.40 ± 0.92^∗^	−34.40 ± 1.57	3.86 ± 0.03
DOX 15 mg/kg+SA 20 mg/kg	224.00 ± 1.58^∗^	197.20 ± 1.82^∗^^#^	−26.80 ± 2.76	4.25 ± 0.08
DOX 15 mg/kg+CAP 30 mg/kg	224.40 ± 1.96^∗^	200.20 ± 1.15^∗^^#^	−24.20 ± 1.88	4.22 ± 0.05

^∗^ denotes significant differences compared to the control group (*p* < 0.05); ^#^ denotes significant differences compared to the DOX group (*p* < 0.05).

**Table 2:** Effects of SA and CAP on serum LDH and CK-MB levels in the different rat groups. Values are expressed as mean ± SEM.

Groups	LDH (U/l)	CK-MB (U/l)
Normal control	199.73 ± 5.37	125.82 ± 2.83
DOX 15 mg/kg	627.42 ± 18.88^∗^	254.36 ± 4.14^∗^
DOX 15 mg/kg+SA 20 mg/kg	217.47 ± 2.84^∗^^#^	157.52 ± 2.98^∗^^#^
DOX 15 mg/kg+CAP 30 mg/kg	211.72 ± 2.66^∗^^#^	152.87 ± 3.47^∗^^#^

^∗^ denotes significant differences compared to the control group (*p* < 0.05); ^#^ denotes significant differences compared to the DOX group (*p* < 0.05).

**Table 3:** Effects of SA and CAP on NO and ET-1 levels.

Group	ET-1 (pg/mg)	NO (*μ*M)
Normal control	128.37 ± 1.72	21.072 ± 1.17
DOX 15 mg/kg	204.77 ± 1.08^∗^	7.003 ± 0.44^∗^
DOX 15 mg/kg+SA 20 mg/kg	145.10 ± 2.02^∗^^#^	13.622 ± 0.27^∗^^**#**^
DOX 15 mg/kg+CAP 30 mg/kg	149.85 ± 2.05^∗^^#^	14.919 ± 0.33^∗^^#^

^∗^ denotes significant differences compared to the control group (*p* < 0.05); ^#^ denotes significant differences compared to the DOX group (*p* < 0.05).

**Table 4:** Effects of sinapic acid (SA) and captopril (CAP) on the levels of lipid peroxidation and antioxidant enzymes. Values are expressed as mean ± SEM.

Group	MDA (nmol/mg)	SOD (U/mg)	GSH (U/mg)	CAT (U/mg)
Normal control	38.01 ± 1.27	35.44 ± 0.82	4.48 ± 0.08	8.12 ± 0.53
DOX 15 mg/kg	124.97 ± 2.67	14.78 ± 0.38	1.76 ± 0.072	2.70 ± 0.21
DOX 15 mg/kg+SA 20 mg/kg	45.92 ± 2.21^∗^	21.33 ± 0.82^∗^^#^	2.52 ± 0.12	5.75 ± 0.22^∗^^#^
DOX 15 mg/kg+CAP 30 mg/kg	43.36 ± 1.52^∗^^#^	18.86 ± 0.68^∗^^#^	2.81 ± 0.08	5.74 ± 0.31^∗^^#^

^∗^ denotes significant differences compared to the control group (*p* < 0.05); ^#^ denotes significant differences compared to the DOX group (*p* < 0.05).

## Data Availability

Data will be available from the corresponding author upon request.

## Abbreviations

SA: Sinapic acid
DOX: Doxorubicin
ROS: Reactive oxygen species
CAP: Captopril
LDH: Lactate dehydrogenase
TNF-*α*: Tumor necrosis factor-*α*
IL-1*β*: Interleukin-1*β*
ET-1: Endothelin-1
NF-*κ*B: Nuclear factor kappa-B
GPx: Glutathione peroxidase
CAT: Catalase
MPO: Myeloperoxidase
ip: Intraperitoneal
CK-MB: Creatinine kinase MB fraction
NO: Nitric oxide
MDA: Malondialdehyde
SOD: Superoxide dismutase.

## Supplementary Materials

Supplementary Materials Graphical abstract (schematic representation of the present investigation).
