# Supplementary figures and images for: Sinapic Acid Ameliorates Oxidative Stress, Inflammation, and Apoptosis in Acute Doxorubicin-Induced Cardiotoxicity via the NF-κB-Mediated Pathway
Source: Biomed Res Int. 2020 Mar 10;2020:3921796. doi: 10.1155/2020/3921796 (PMC7085847; doi:10.1155/2020/3921796)

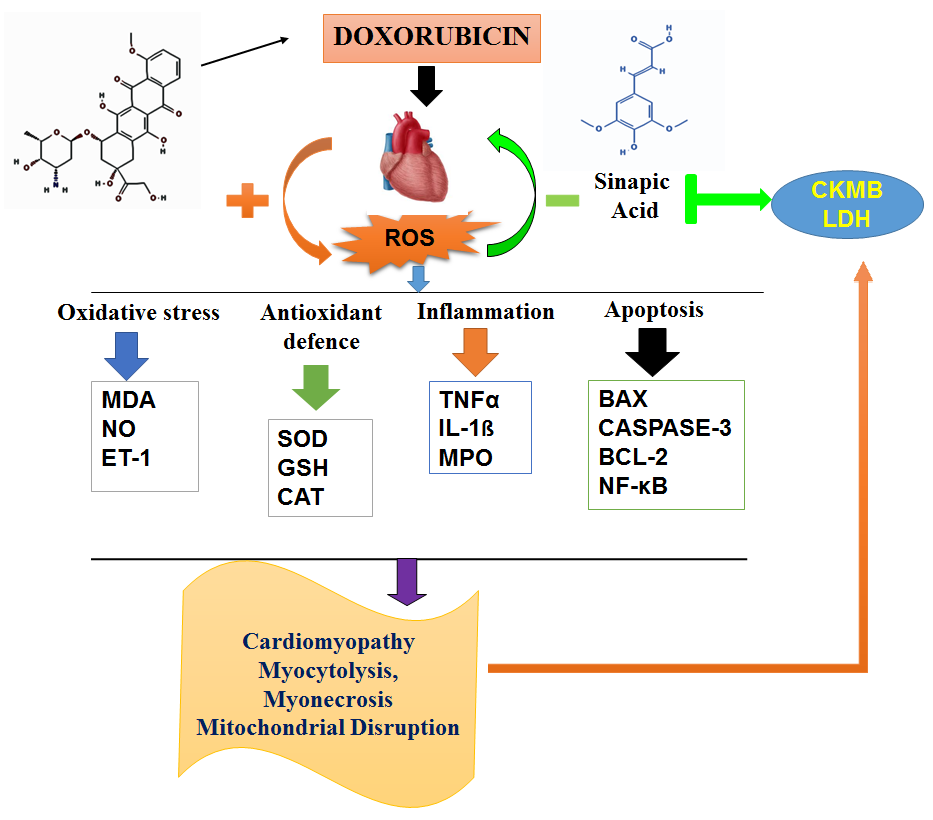


**Figure**: Graphical Abstract (Schematic representation of the present investigation).

Supplement: Supplementary Materials — Graphical abstract (schematic representation of the present investigation). [file 3921796.f1.docx]
